# Supplementary material for: A first-in-class inhibitor of Hsp110 molecular chaperones of pathogenic fungi
Source: Nat Commun. 2023 May 12;14:2745. doi: 10.1038/s41467-023-38220-2 (PMC10182041; doi:10.1038/s41467-023-38220-2)
Supplement: Supplementary file 1 — Supplementary Information [file 41467_2023_38220_MOESM1_ESM.pdf]

# Figure S1

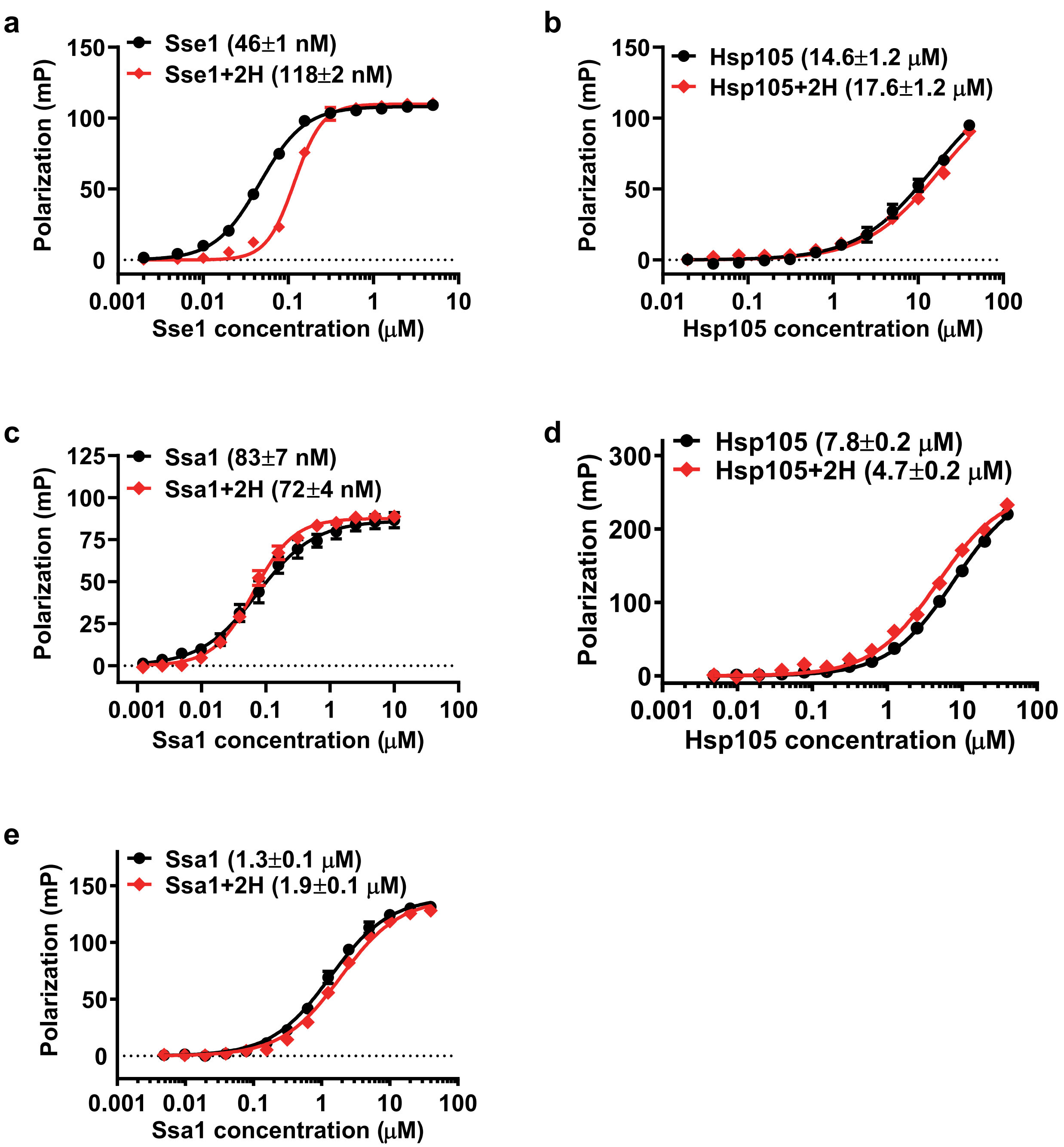


**Figure S1 – 2H showed limited impact on the binding of ATP or peptide substrates to Ssa1, Hsp105, and Sse1.**
**(a**, **b**, **c)** ATP binding to Sse1 (**a**), Hsp105 (**b**), and Ssa1 (**c**). ATP-FAM was used to determine ATP binding following the same fluorescence polarization assay as for Msi3 in **Fig. 1c**. Data are presented as mean values +/- SEM (n=3 (**a**), 4 (**b**), and 6 (**c**) independent experiments using different protein purifications).
**(d**, **e)** Peptide substrate binding to Hsp105 (**d**) and Ssa1 (**e**). The same fluorescent polarization assay was carried out as in **Fig. 2b**. The TRP2-181 peptide was used for Hsp105. For Ssa1, the NR peptide, a well-established peptide substrate for Hsp70s, was used. Data are presented as mean values +/- SEM (n=4 (**d**) and 3 (**e**) independent experiments using different protein purifications). Dissociation constant ($K_d$) are listed in parentheses.
Source data are provided as a Source Data file for all the panels.

# Figure S2

| | Msi3 | Hsp105 |
|---|---|---|
| **Hsp105** | **38.5** | **-** |
| **Apg-1** | **36.7** | **59.2** |
| **Apg-2** | **37.5** | **64.3** |
| **Sse1** | **64.0** | **39.5** |
| **Hsp110-*auris*** | **73.3** | **37.7** |
| **Hsp110-*glabrata*** | **63.7** | **39.2** |
| **Hsp110-*parapsilosis*** | **83.9** | **38.2** |
| **Hsp110-*tropicalis*** | **88.7** | **39.5** |

**Figure S2 – The pairwise sequence identifies among selective Hsp110s.**
There are three known human Hsp110s: Hsp105, Apg-1, and Apg-2. There are four common pathogenic *Candida* species: *C. auris, C. glabrata, C. parapsilosis, and C. tropicalis*. Each of these species has a hypothetical Hsp110: Hsp110-*auris,* Hsp110-*glabrata,* Hsp110-*parapsilosis,* and Hsp110-*tropicalis*, respectively.

# Figure S3

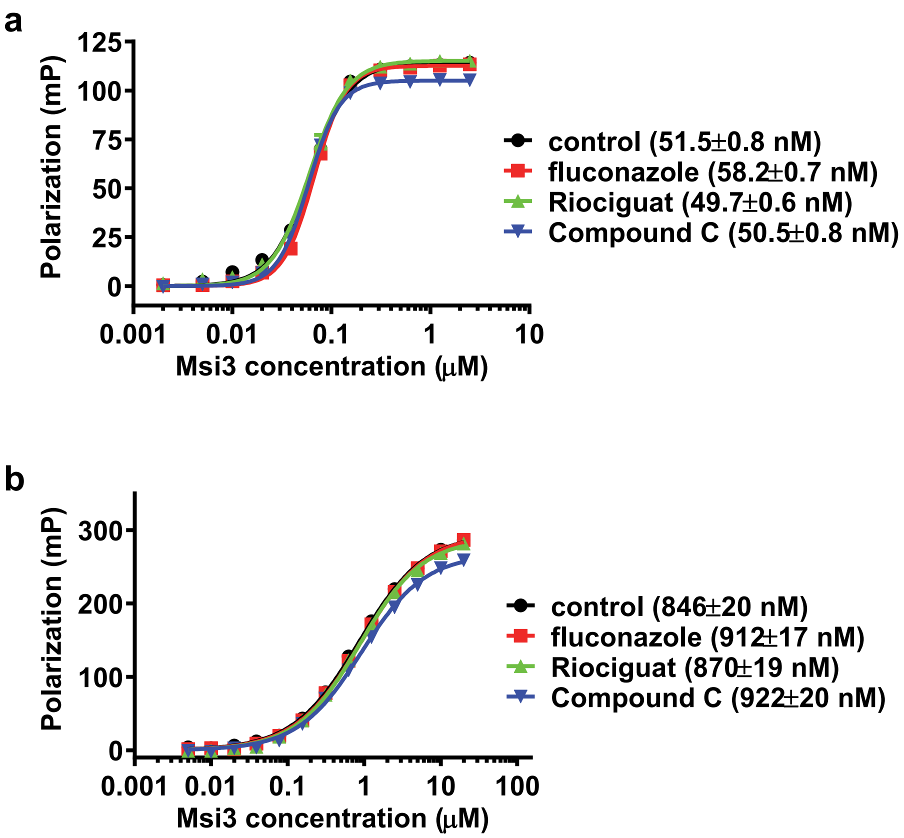


**Figure S3 – The effect of fluconazole, Compound C, or Riociguat on the binding of ATP (A) and TRP2-181 peptide (B) to Msi3.**

The same fluorescence polarization assays as shown in **Fig. 1c** (for ATP binding) and **2b** (for binding to the TRP2-181 peptide) were carried out. Data are presented as mean values +/- SEM (n=3-4 independent experiments with more than two separate protein purifications). Dissociation constants ($K_d$) were calculated and are listed in parentheses. Source data are provided as a Source Data file.

## Figure S4

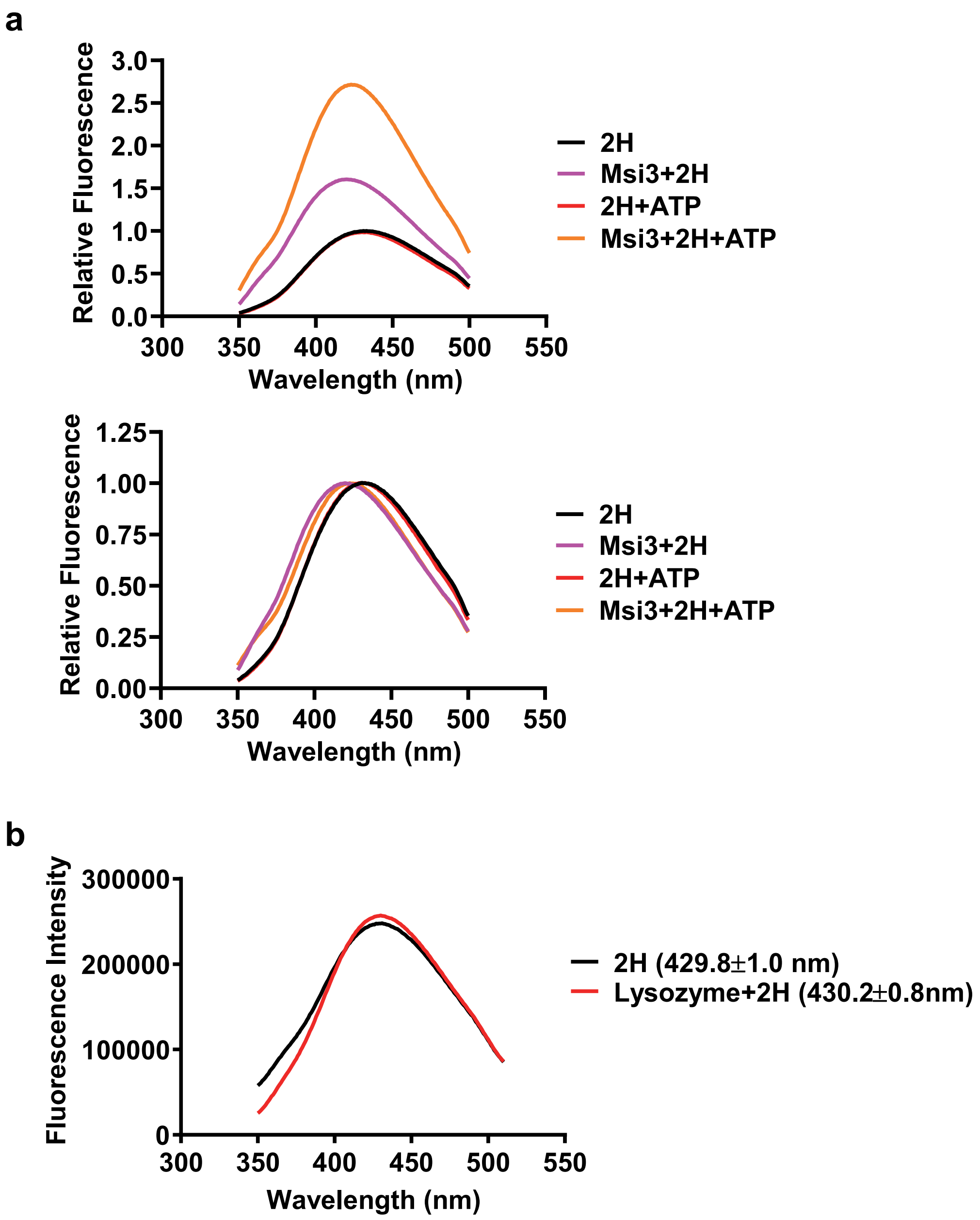


**Figure S4 – Fluorescence spectra of 2H.**
**(a)** The relative fluorescence of 2H from **Fig. 1d**. Top panel: to show the comparative intensity difference, the relative fluorescence was plotted by setting the peak value of 2H alone (black line) as 1. Bottom panel: to show the shifts, the relative fluorescence of each spectrum was calculated by setting the peak value of the corresponding spectrum as 1.
**(b)** Lysozyme showed little influence on the fluorescence spectra of 2H. The fluorescence spectra of 2H were collected in the presence and absence of lysozyme, using the same method as in **Fig. 1d**. The peak wavelengths are listed in parentheses. Source data are provided as a Source Data file.

# Figure S5

| | $k_{off}$ (x10$^{-3}$/sec) |
|---|---|
| **buffer** | **0.370±0.063** |
| **Buffer+2H** | **0.478±0.083** |
| **ATP** | **0.609±0.049** |
| **ATP+2H** | **0.596±0.048** |
| **Msi3** | **0.716±0.062** |
| **Msi3+2H** | **0.694±0.033** |
| **Msi3+ATP** | **5.000±0.043** |
| **Msi3+ATP+2H** | **4.030±0.032** |

**Figure S5 – Release kinetics ($k_{off}$) of ATP-FAM from Ssa1**.
$k_{off}$ values were calculated from **Fig. 2c**.

# Figure S6

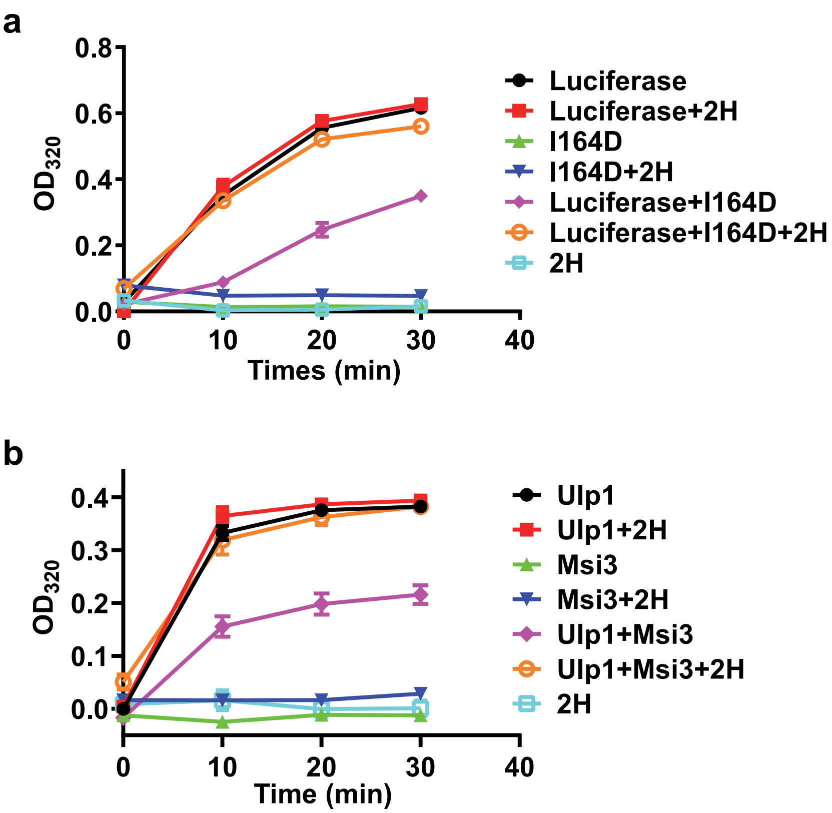


**Figure S6 – 2H inhibited the holdase activity of Msi3 proteins.**
**(a)** The holdase activity of the Msi3-I164D mutant protein was inhibited by 2H. The assay was carried out in the same way as for the WT Msi3 in **Fig. 3a**.
**(b)** 2H inhibited the holdase activity of Msi3 when Ulp1 was used as a substrate. The aggregation of Ulp1 during heat treatment at 42 °C was monitored using $OD_{320}$–similarly to the use of luciferase as a model substrate in **Fig. 3a**.
Data in both panels are presented as mean values +/- SEM (n=6 independent experiments with more than two different protein purifications). Source data are provided as a Source Data file.

# Figure S7

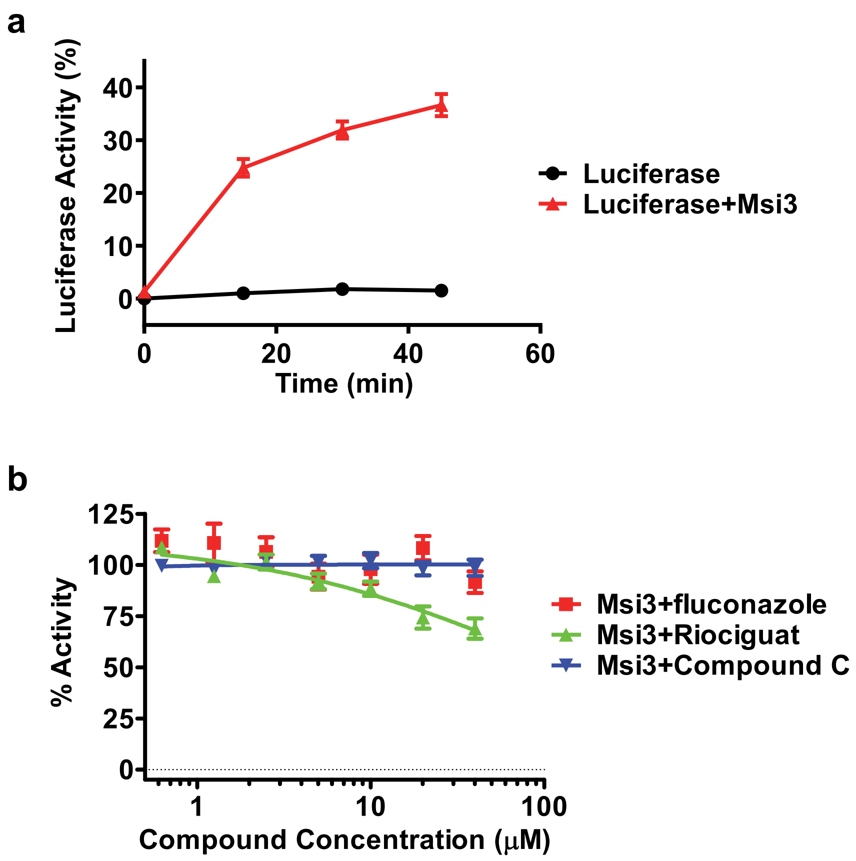


**Figure S7 – The effects of selected compounds on the holdase activity of Msi3.**

**(a)** The holdase activity of Msi3 was assayed by measuring the ability of luciferase to refold. After heating at 42 °C for 10 min, luciferase lost its ability to refold in the presence of the Ssa1/Msi3/Ydj1 chaperone machinery due to the aggregation caused by heating (black circles). In contrast, including Msi3 during the 10-min incubation at 42 °C resulted in significant refolding of luciferase by the Ssa1/Msi3/Ydj1 chaperone system (red triangle), indicating the holdase activity of Msi3 during the 42 °C incubation. The activity of luciferase during the refolding by the Ssa1/Msi3/Ydj1 chaperone machinery was plotted, and the activity of luciferase without heat treatment was set as 100%. Data are presented as mean values +/- SEM (n=5 independent experiments).

**(b)** Fluconazole, Compound C, and Riociguat each exhibited limited impact on the holdase activity of Msi3. The holdase assay was carried out in the same way as in **Fig. 3b**. The activity of luciferase at 30-min refolding was measured, and relative activities were plotted by setting the activity in the absence of any compounds as 100%. Data are presented as mean values +/- SEM (n=5-6 independent experiments with at least two separate protein purifications).

Source data are provided as a Source Data file for both panels.

## Figure S8

a

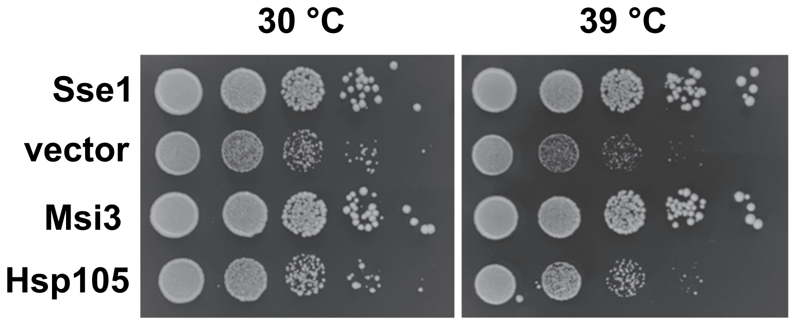


b

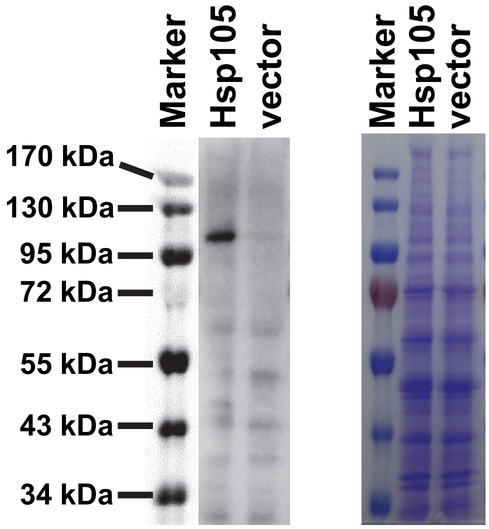


**Figure S8 – Hsp105 failed to functionally replace Sse1 in *S. cerevisiae*.**
**(a)** Growth tests. After transforming the *S. cerevisiae sse1Δ* strain with a pRS313 plasmid carrying either *SSE1*, *MSI3*, or *Hsp105*, serial dilutions of yeast cultures were spotted on plates containing yeast synthetic medium without histidine (His Dropout). Pictures were taken after growing at 30 or 39 °C. Empty vector was used as a negative control. Representative plates are shown.
**(b)** Hsp105 showed significant expression in yeast. The expression of Hsp105 was analyzed by Western-blot using an antibody against Hsp105 (AF4029SP and SA510314 for primary and secondary antibodies, respectively, from Fisher Scientific; left panel). Yeast transformed with the empty vector was used as a negative control. The same amount of yeast lysates was loaded to SDS-PAGE and stained with Coomassie blue (right panel) to show equivalent loading of samples.
Source data are provided as a Source Data file for both panels.

# Figure S9

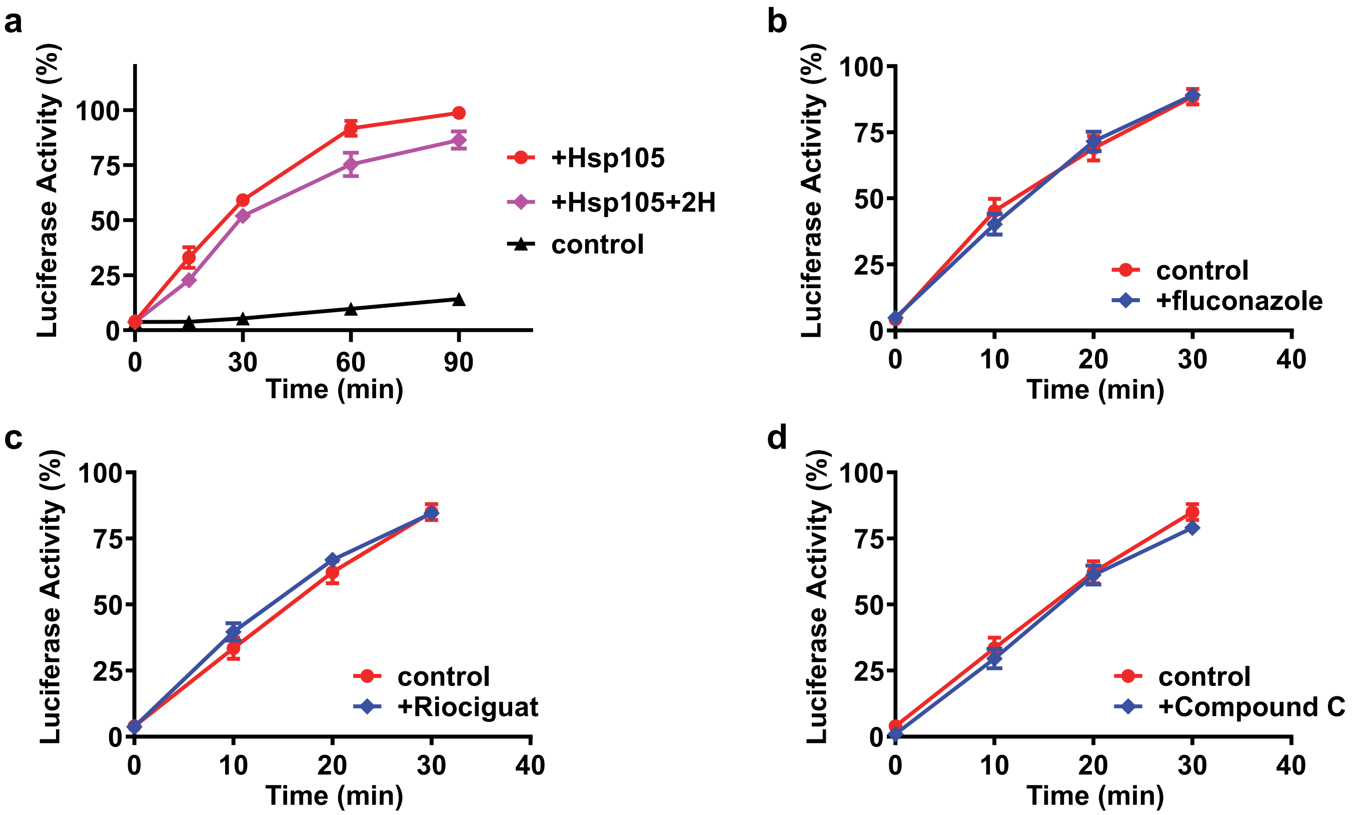


**Figure S9 – Effects of compounds on the refolding activity of Hsp70 chaperone systems.**

**(a)** 2H exhibited limited inhibition on the refolding activity of the human Hsp70/Hsp105/HDJ2 chaperone machinery. The same refolding assay was conducted as in **Fig. 3c**, but human chaperones were used in place of the homologous yeast chaperones. The reaction without Hsp105 (i.e., Hsp70/HDJ2) was used as a negative control.

**(b**, **c**, and **d)** Neither Fluconazole (**b**), Riociguat (**c**), nor Compound C (**d**) showed significant impact on the refolding activity of the Ssa1/Msi3/Ydj1 chaperone system. The refolding assay using luciferase as a substrate was carried out in the same way as in **Fig. 3c**. Control: no compound added.

Data in all panels are presented as mean values +/- SEM (n=7 independent experiments with more than two different protein purifications). Source data are provided as a Source Data file.

## Figure S10

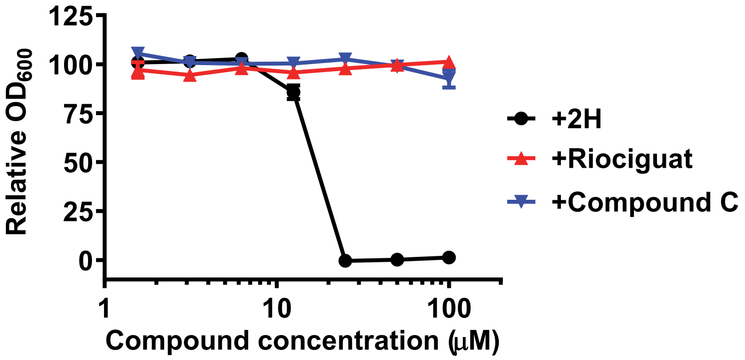


**Figure S10 – Neither Riociguat nor Compound C showed a significant impact on the growth of SC5314.**

Tests were carried out in the same way as those on 2H in **Fig. 4a**. The same data on 2H shown in **Fig. 4a** was plotted and used for comparison. The relative $OD_{600}$ was calculated by setting the $OD_{600}$ values of the growth control as 100%. Data are presented as mean values +/- SEM (n=6-8 independent experiments). Source data are provided as a Source Data file.

## Figure S11

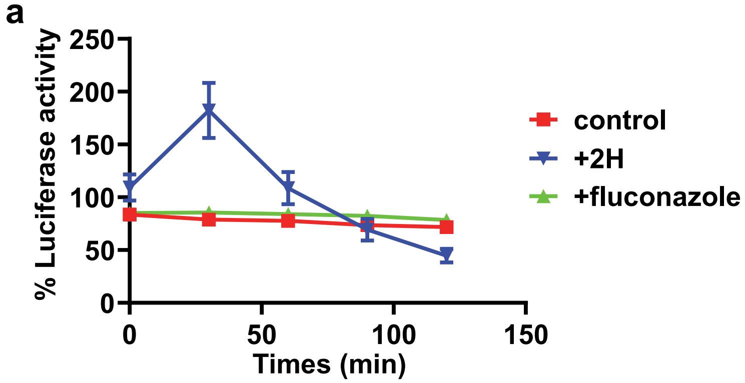


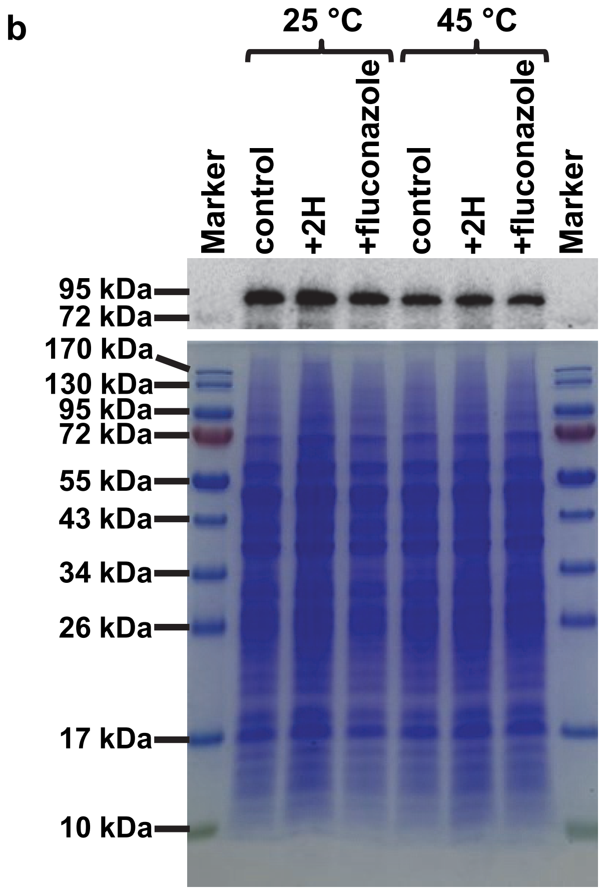


**Figure S11 – The effect of 2H on the *in vivo* activity and stability of luciferase expressed in *S. cerevisiae*.**

**(a)** *In vivo* luciferase activity at 25 °C. The experiment was carried out in the same way as in **Fig. 5a**, except that denaturation at 45 °C was not applied. The transient increase of the luciferase activity at the 30-min time points for the cells treated with 2H may be due to some unknown effect of 2H. Data are presented as mean values +/- SD (n=4-5 independent experiments).

**(b)** Neither 2H nor fluconazole significantly affected the stability of luciferase. Luciferase expression was induced for 1 hour, after which cycloheximide was added to stop further protein expression. A 45 °C heat shock treatment was applied, the culture was then split, and a treatment with either 2H or fluconazole was applied (the 45 °C samples). The levels of luciferase were analyzed using Western blot with an antibody against luciferase. A no-compound treatment was used as a control. The 25 °C samples were controls without the 45 °C heat shock and were performed in the same way as in **a**.
Source data are provided as a Source Data file for both panels.

# Figure S12

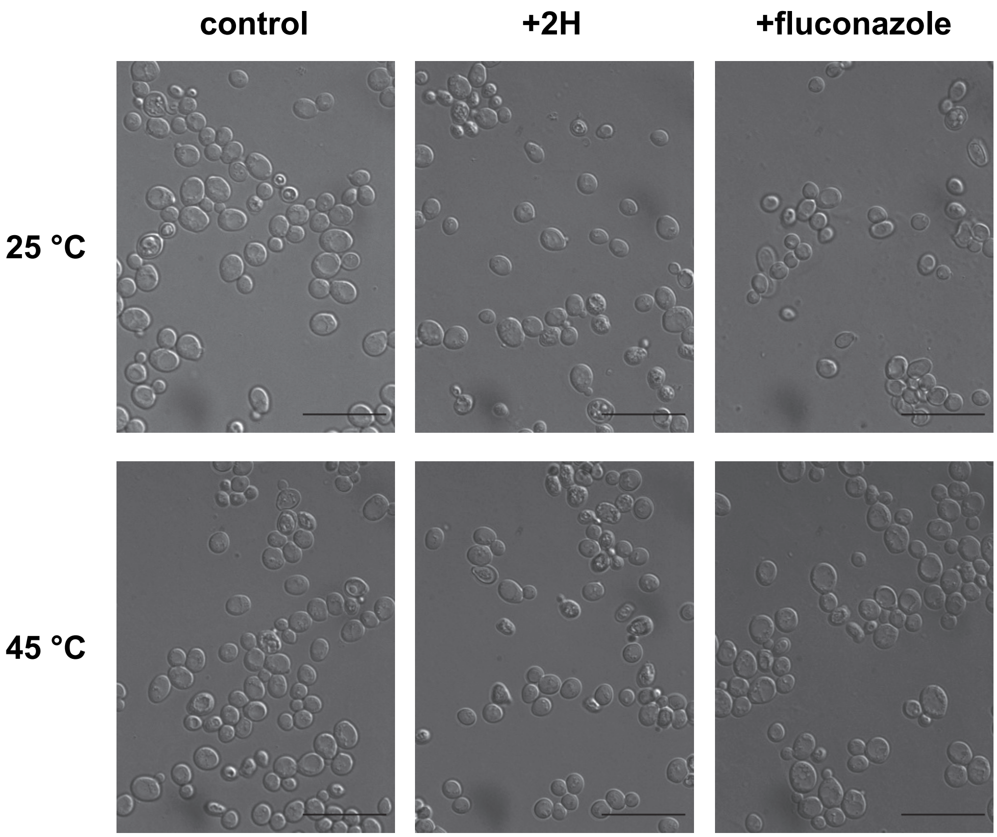


**Figure S12 – *S. cerevisiae* cells remained largely intact after 2-hour treatment with 2H or fluconazole.**
The images of *S. cerevisiae* cultures were captured under a light microscope. Representative images are shown. The scale bar is 20 μm. Source data are provided as a Source Data file.

# Figure S13

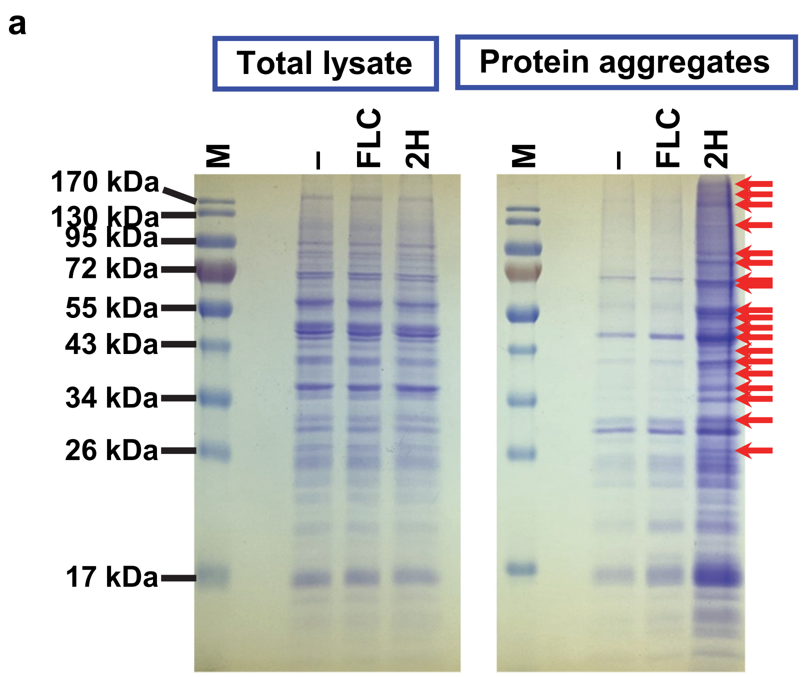


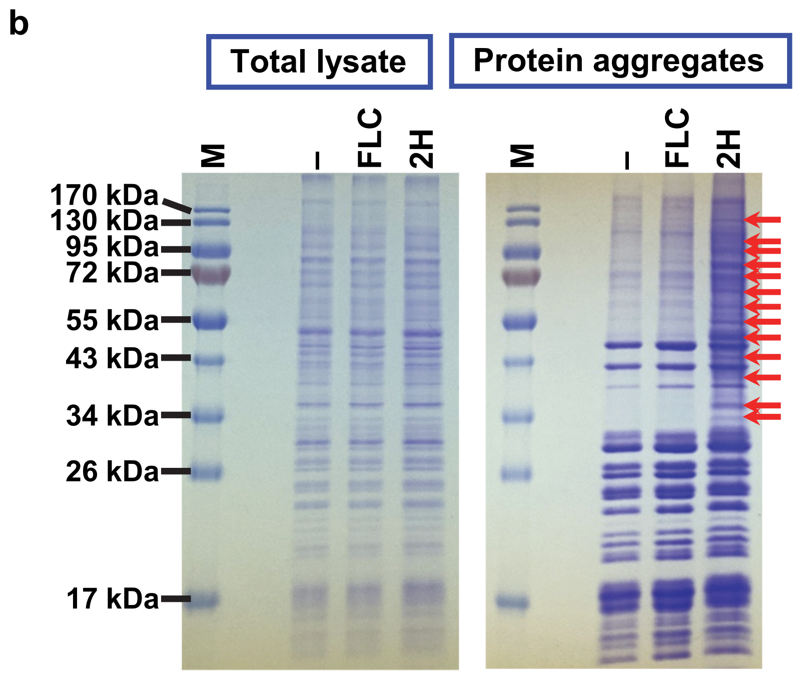


**Figure S13 – 2H treatment resulted in enhanced protein aggregation in both *S. cerevisiae* (a) and *C. albicans* (b).**
Assays were conducted similarly to those in **Fig. 5b**, but without the 45 °C heat treatment. Final concentrations: 2H = 80 μM for **a** and 100 μM for **b**; fluconazole (FLC) = 100 μM. "–" corresponds to the control without the addition of any compound. Red arrows indicate selected unique or enriched bands in the samples treated with 2H compared to the controls and total lysates. Source data are provided as a Source Data file for both panels.

# Figure S14

a. Ergosterol biosynthesis

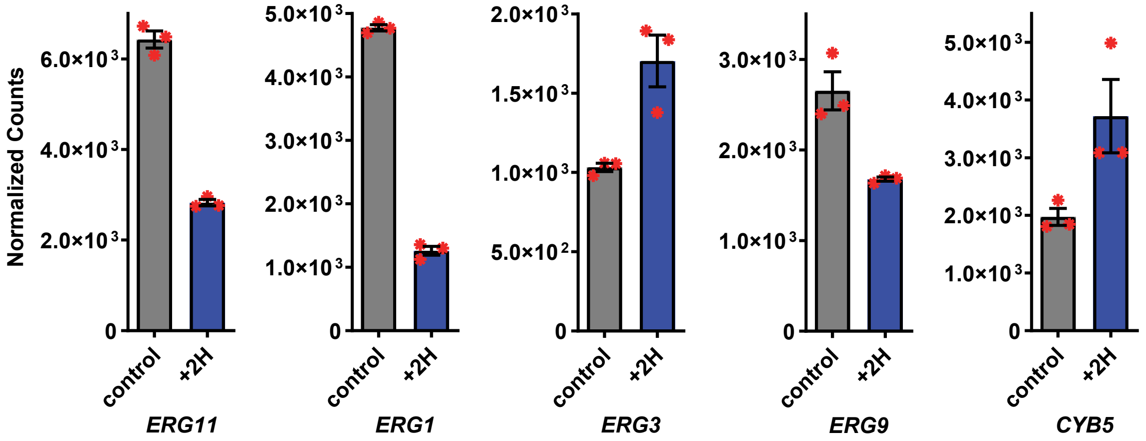


b. Multidrug efflux pump and pleiotropic efflux transporters

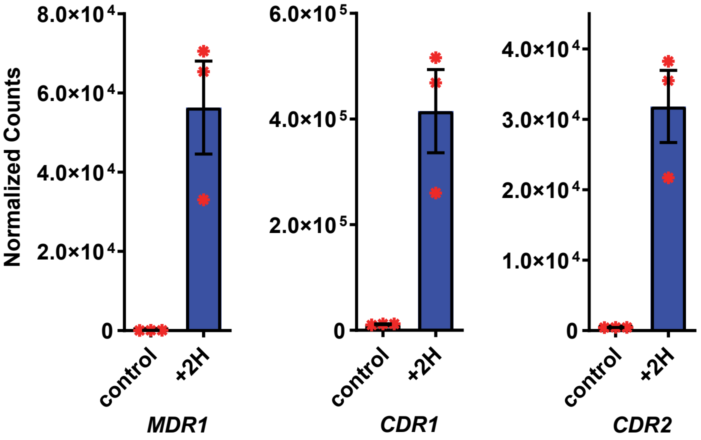


**Figure S14 – Transcriptional changes of selected genes upon 2H treatment.** The plots were prepared in the same way as in **Fig. 6a**. Normalized counts of transcription were used. The control was without 2H treatment.

**(a)** Transcriptional changes of selected genes in ergosterol biosynthesis. The expression of these genes was reported to be up-regulated upon fluconazole treatment.

**(b)** *MDR1* (a multidrug efflux pump) and *CDR1* and *CDR2* (two pleiotropic efflux transporters) were up-regulated upon 2H treatment. These genes confer resistance to numerous chemicals.

Data in all plots are presented as mean values +/- SEM (n=3 independent experiments). Individual data points are shown in red. Source data are provided as a Source Data file.

# Figure S15

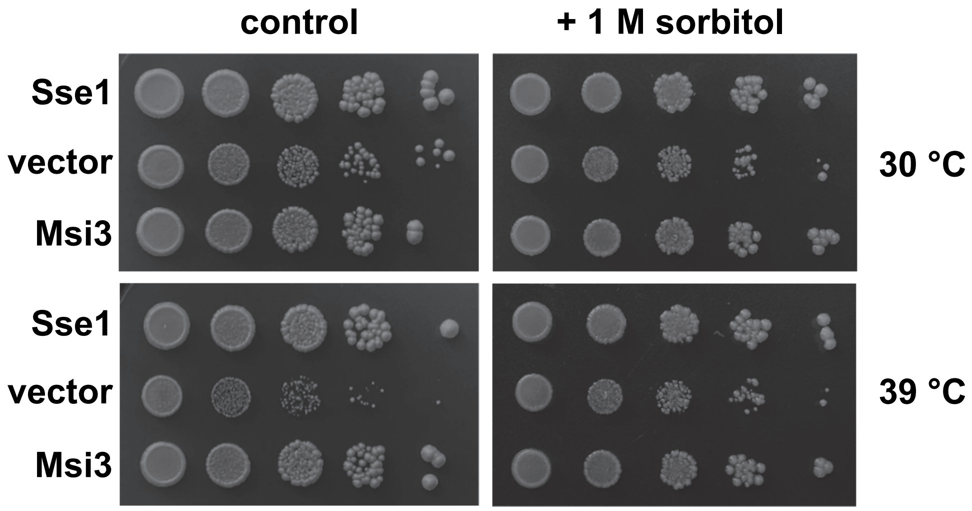


**Figure S15 – High osmolality partially suppressed the temperature-sensitive phenotype of our *S. cerevisiae sse1Δ* strain.**
The growth tests were performed in the same way as in **Fig. S8** on His Dropout plates alone (control, left panels), or His Dropout plates containing 1 M sorbitol to induce high osmolality (right panels). Representative plates grown at 30 and 39 °C are shown. Source data are provided as a Source Data file.

# Figure S16

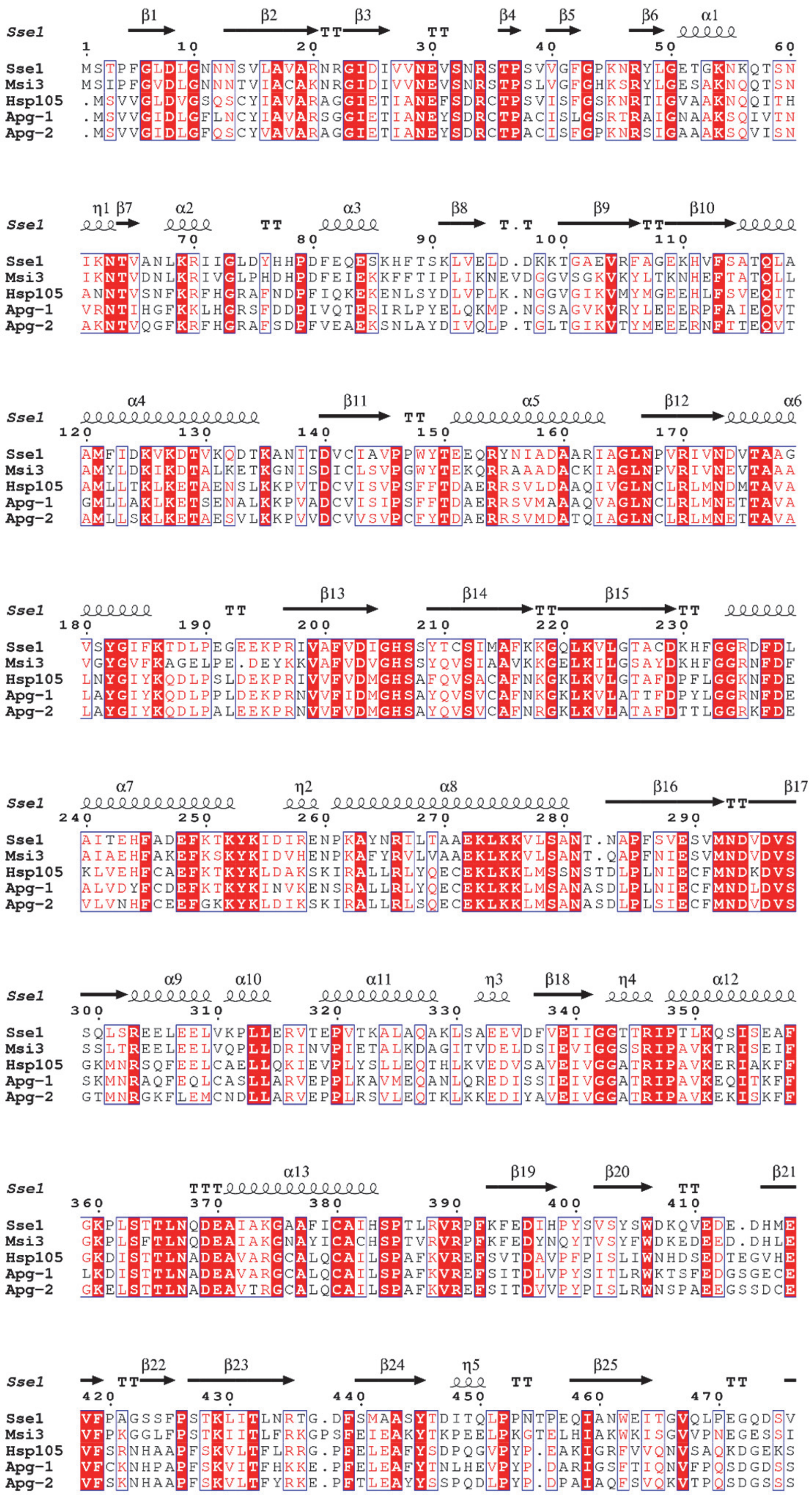

## Figure S16

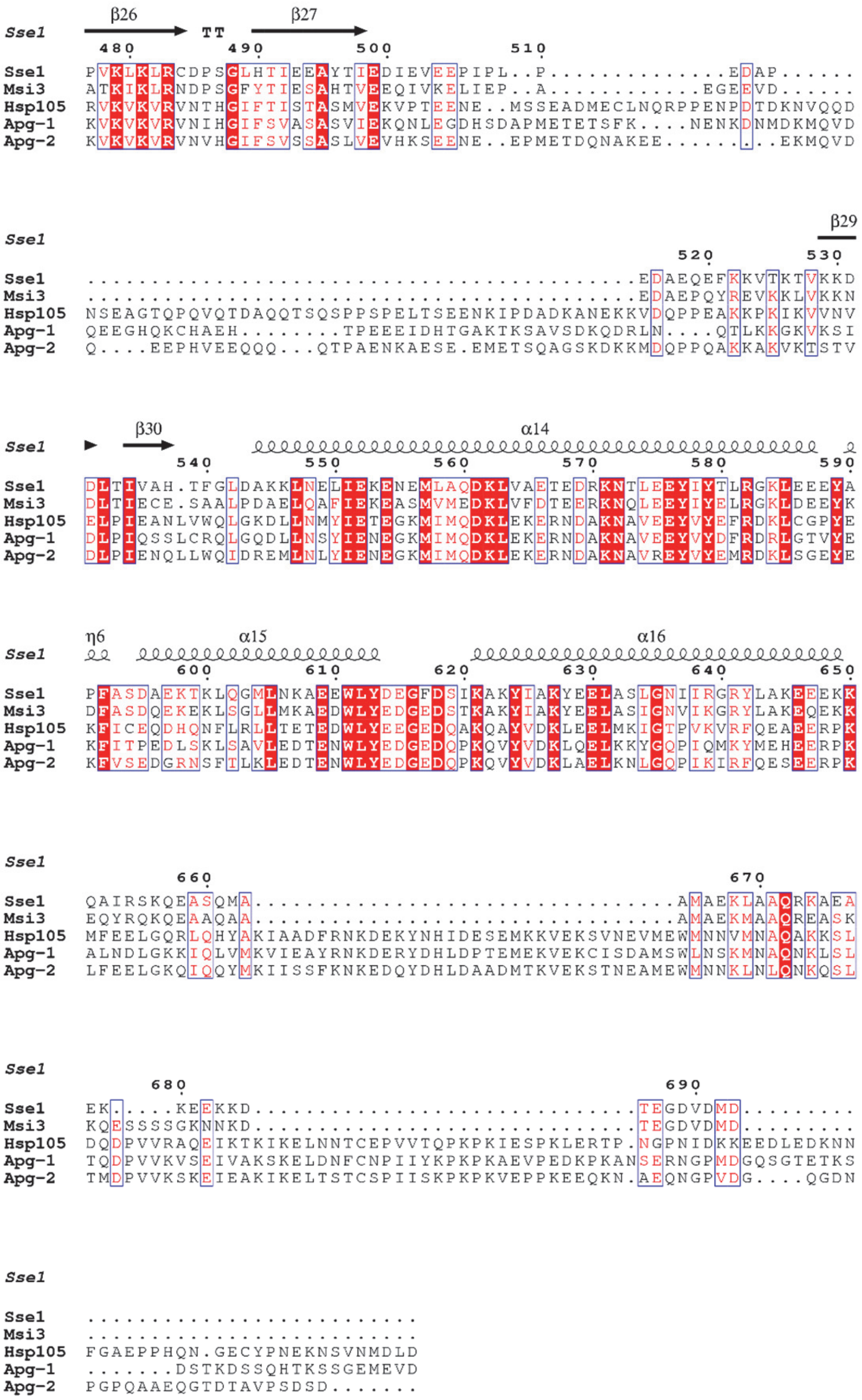


**Figure S16 – The sequence alignment.**
Sse1 and Msi3 are from *S. cerevisiae* and *C. albicans*, respectively. The three known human Hsp110s are: Hsp105, Apg-1, and Apg-2. The secondary structural elements of the Sse1 structure (pdb: 2QXL) are listed at the top of the sequence alignment.

# Table S1

| genes | class | location | major known function | change |
|---|---|---|---|---|
| *MSI3* | Hsp110 | Cytosol | protein folding, import, preventing and solubilizing protein aggregation | 3.38±0.34 |
| *SSA1* | Hsp70 | Cytosol | protein folding, import, preventing and solubilizing protein aggregation | 3.93±0.36 |
| *YDJ1* | Hsp40 | Cytosol | protein folding, import, preventing and solubilizing protein aggregation | 4.53±0.09 |
| *JJJ3* | Hsp40 | Cytosol | protein import into ER | 9.28±2.96 |
| *CAJ1* | Hsp40 | Cytosol | protein import, assembly, and disassembly | 10.16±2.36 |
| *FES1* | NEF | Cytosol | protein folding, degradation | -1.17±0.08 |
| *HSF* | HSF | Cytosol | stress response | 2.71±0.63 |
| *HSP104* | Hsp100 | Cytosol | solubilizing protein aggregation | 2.22±0.13 |
| *HSP90* | Hsp90 | Cytosol | activation of regulatory and signaling proteins, protein folding | 1.18±0.46 |
| *CCT1** | TRiC/CCT | Cytosol | folding of newly translated proteins | 1.38±0.01 |
| *SSB1* | Hsp70 | Cytosol | folding of newly translated proteins | 1.54±0.48 |
| *SSZ1* | Hsp70 | Cytosol | folding of newly translated proteins | 1.59±0.18 |
| *ZUO1* | Hsp40 | Cytosol | folding of newly translated proteins | 2.10±0.08 |
| *KAR2* | Hsp70 | ER | protein folding in ER | 1.58±0.13 |
| *SSC1* | Hsp70 | Mitochondria | protein import and folding in mitochondria | 1.86±0.49 |
| *HSP60* | Hsp60 | Mitochondria | protein folding in mitochondria | 1.68±0.21 |

**Table S1 – The 2H-induced transcriptional changes of Msi3 and related chaperones.**
The change in transcription (right-most column) is shown as the ratio of the 2H-treated sample over the control for up-regulated genes, or the ratio of the control over the 2H-treated sample for down-regulated genes with a negative sign. The mean values ± SEM are listed (n=3 independent experiments).
*TRiC/CCT is represented by CCT1, one of the eight subunits of the TRiC/CCT complex. The other 7 subunits showed a similar change in transcription.
